# The role of individual differences in resistance to persuasion on memory for political advertisements

**DOI:** 10.3389/fpsyg.2023.1196209

**Published:** 2023-08-09

**Authors:** Stuart S. Miller, John P. Hutson, Megan L. Strain, Tim J. Smith, Maria Palavamäki, Lester C. Loschky, Donald A. Saucier

**Affiliations:** ^1^Department of Psychological Sciences, Kansas State University, Manhattan, KS, United States; ^2^Psychology, University of Nebraska at Kearney, Kearney, NE, United States; ^3^Department of Psychological Sciences, University of London Birkbeck College, London, United Kingdom; ^4^Department of Media, Aalto University, Otakaari, Finland

**Keywords:** social vigilantism, need for cognition, resistance to persuasion, attitudes, memory, political advertisements

## Abstract

When people see political advertisements on a polarized issue they take a stance on, what factors influence how they respond to and remember the adverts contents? Across three studies, we tested competing hypotheses about how individual differences in *social vigilantism* (i.e., attitude superiority) and *need for cognition* relate to intentions to resist attitude change and memory for political advertisements concerning abortion. In Experiments 1 and 2, we examined participants’ intentions to use resistance strategies to preserve their pre-existing attitudes about abortion, by either engaging against opposing opinions or disengaging from them. In Experiment 3, we examined participants’ memory for information about both sides of the controversy presented in political advertisements. Our results suggest higher levels of social vigilantism are related to greater intentions to counterargue and better memory for attitude-incongruent information. These findings extend our understanding of individual differences in how people process and respond to controversial social and political discourse.

## 1. Introduction

Nothing limits intelligence more than ignorance; nothing fosters ignorance more than one’s own opinions; nothing strengthens opinions more than refusing to look at reality.—Sheri S. Tepper

Opposing opinions about political and social issues often provoke sharp and contentious debate. It seems impossible to escape confrontations with opponents when people voice their positions on political or social issues. But when confrontation occurs, people may decide to engage the opponent by investing attention and effort to debate, or they may disengage from the opponent by diverting attention and conserving effort. We examined these decisions by investigating individual differences related to how people respond to attempted persuasion. Specifically, when faced with highly controversial social issues in political advertisements, do people engage in active resistance to attitude challenges, or engage in selective exposure away from them? Additionally, do these strategies for resisting persuasion affect memory for pro-attitudinal vs. counter-attitudinal arguments?

Social psychologists have examined individual difference factors that influence how receptive vs. resistant people are to attitude change attempts for decades (for reviews, see Petty and Wegener, 1998; Crano and Crislin, 2006). Stronger attitudes are harder to change (Krosnick et al., 1993; Eagly and Chaiken, 1995; Krosnick and Petty, 1995; Pomerantz et al., 1995; Zuwerink and Devine, 1996; Jacks and Devine, 2000; Jacks and Cameron, 2003; Visser et al., 2003; Skitka et al., 2005; Visser et al., 2006; Saucier et al., 2014), and challenges to strongly held attitudes provoke people to resist these attempts either by engaging with the persuasion attempt or by disengaging from it. Additionally, the strategies people use to resist such attempts vary with their individual differences in *need for cognition* (Cacioppo and Petty, 1982; Cacioppo et al., 1986) and *social vigilantism* (Saucier and Webster, 2010). In contrast to much previous work, here we do not focus on attitude change, but instead we investigate how individual differences in social vigilantism and need for cognition predict people’s strategies to resist persuasion and their memory for the contents of the persuading arguments.

### 1.1. Strategies to resist persuasion

When people are motivated to resist persuasion, and preserve their pre-existing attitudes, they may use various strategies to actively or passively counter the specific persuasion attempt or attitude challenge. While people may use several strategies to resist persuasion (see Jacks and Cameron, 2003; Saucier et al., 2014), we focus on two: the active strategy of counterarguing by which people attack the opposing information, and the passive strategy of selective exposure by which people withdraw from and avoid the opposing information. Importantly, these two strategies involve fundamentally different choices to either engage with or avoid opposing information, which have important implications for later memory for the persuasive material.

### 1.2. Need for cognition

People higher in need for cognition are less likely to be persuaded by peripheral cues, such as the number of arguments (regardless of their quality) or the physical attractiveness of speakers (for a review, see Cacioppo et al., 1996), and prefer, are more receptive to, and have a better memory for cognitive vs. emotional appeals (Haddock et al., 2008). Importantly, higher levels of need for cognition are related to greater resistance to attitude change attempts (Haugtvedt and Petty, 1992) and therefore may also be related to greater counterarguing as a resistance strategy. Additionally, people higher in need for cognition may be more likely to engage with the information presented about an issue (and therefore less likely to engage in selective exposure), more likely to pay attention to it, and be more likely to recall it later (Peltier and Schibrowsky, 1994). Because need for cognition is related to the tendency to engage information (as opposed to ignoring it), selective exposure may be less likely to occur regardless of whether the information supports or opposes people’s pre-existing attitudes about an issue. Existing findings are inconclusive on whether need for cognition is associated with greater tendencies to engage in selective exposure (e.g., Westerwick et al., 2017; Knobloch-Westerwick et al., 2020; Ryu and Vargas, 2021) or lesser tendencies to do so (e.g., Tsfati and Cappella, 2005).

### 1.3. Social vigilantism

Social vigilantism is the tendency for people to believe their own attitudes are superior to others’ attitudes, to resist persuasion attempts and attitude challenges, and to attempt to impress their own attitudes on others (Saucier and Webster, 2010; Saucier et al., 2017). Higher levels of social vigilantism are related to greater resistance to persuasion. Specifically, people higher in social vigilantism have more extreme attitudes and show greater use of various resistance strategies—particularly counterarguing—in response to attitude challenges about a range of socially controversial topics (Saucier and Webster, 2010; Raimi and Leary, 2014; Saucier et al., 2014; Maki and Raimi, 2017; O’Dea et al., 2018). Counterarguing involves directly engaging and trying to defeat an opposing argument, and is a commonly used and effective technique for resisting persuasion (Cameron et al., 2002; Jacks and Cameron, 2003). Social vigilantism has consistently been related to counterarguing, but has been inconsistently related to using passive strategies to resist persuasion, with some studies finding that social vigilantism is positively correlated with selective exposure (O’Dea et al., 2018), but others not (Saucier et al., 2014).

Because it is still unclear whether social vigilantism and need for cognition are related to selective exposure to resist persuasion for strongly held attitudes, we investigated this question in the current study. We focused on these two individual difference constructs in particular because of their relevance to engaging with, and processing of, persuasive messages. Social vigilantism is important for understanding how belief superiority and motivations to impress self-professed superior attitudes upon others relate to counterarguing. Need for cognition is relevant for predicting whether people will engage in selective exposure because need for cognition represents individuals’ tendencies to engage with information. To further understand how these individual differences relate to counterarguing and selective exposure, we additionally examined the consequence of these effects on memory for the provocative content. The objective of any political advertising is to first get you to attend to the content, then remember it and eventually incorporate the content into your own beliefs. As we were not examining attitude change for strongly held beliefs in our research, we stop at the earlier stage of analyzing whether memory for content is selective and congruent with participants’ preexisting beliefs.

### 1.4. Linking resistance strategies to subsequent memory

Because of the likely differences in cognitive processing engendered by counterarguing vs. selective exposure strategies, there are strong reasons to hypothesize links between the strategies people report using in response to attitude-congruent vs. -incongruent information and their subsequent memory for it. People remember what they attend to in their environment (Loftus, 1972; Hollingworth and Henderson, 2002; Tatler et al., 2005; Zelinsky and Loschky, 2005; Pertzov et al., 2009). Counterarguing and selective exposure strategies likely differ in attention, thus also in memory. Specifically, in order to counterargue, one must attend to and process information, thus encoding memory for it.

#### 1.4.1. Selective exposure and memory

Theories of selective exposure argue that people will avoid or tune out attitude-incongruent information. Memory experiments have found that people tend to have worse memory for attitude-incongruent information (Eagly and Chaiken, 1995). However, there are several important considerations concerning these selective exposure memory effects. Meta-analyses show that selective exposure effects on memory across studies are fairly weak (Eagly and Chaiken, 1995). However, these effects become stronger when individuals’ attitude strength is included as a moderating variable, with selective exposure effects on memory being more common for people with weakly held, but highly partisan attitudes (Eagly and Chaiken, 1995). Conversely, people with strongly held beliefs tend not to show selective exposure effects, but rather use resistance strategies that increase their engagement with attitude-incongruent information. Critically, although people clearly engage in selective exposure, effects on memory are not entirely dependent on attitude congruence. Instead, moderating variables such as attitude strength, propensity to counterargue, and belief superiority are also important for understanding how people engage attitude-incongruent information (Albarracín and Mitchell, 2004; Brannon et al., 2007).

### 1.5. Overview of the current experiments

The current research extends that on selective exposure and memory in two important ways. First, we test whether social vigilantism and need for cognition are related to the strategies people use to resist persuasion for strongly held attitudes. Second, we test whether these individual differences relate to memory for attitude-relevant information as evidence of engaging with the contents of persuasion attempts (i.e., counterarguing) or ignoring them (i.e., selective exposure).

Across three experiments, participants viewed a series of political ads and completed attitude and persuasion questionnaires (Experiments 1 and 2), or memory measures (Experiment 3). We used both controversial and non-controversial ads to manipulate attitude congruence (congruent, incongruent, and neutral). We used the topic of abortion because: (1) it was a familiar topic to the participant pool used; (2) people typically have strong attitudes about abortion; and (3) pilot studies showed a bimodal distribution with a fairly even proportion of participants with highly pro-life vs. pro-choice attitudes.

In Experiments 1 and 2, participants viewed either a pro-choice or a pro-life ad and reported their intentions to engage in counterarguing and selective exposure. In Experiment 3, participants watched the videos and completed memory tests about the content of the videos. We examined how need for cognition and social vigilantism were related to (a) participants’ intentions to respond with counterarguing or selective exposure (Experiments 1 and 2), and (b) participants’ memory (Experiment 3).

## 2. Experiment 1

In Experiment 1, we tested two hypotheses: the *social vigilantism hypothesis* and the *need for cognition hypothesis*. Both hypotheses predict people higher in that individual difference variable, either social vigilantism or need for cognition, should report stronger intentions to engage in counterarguing. For selective exposure, the need for cognition hypothesis predicts that higher levels of need for cognition will be associated with weaker intentions to engage in selective exposure in response to attitude-*incongruent* political ads about the issue of abortion. However, because past research has found inconsistent relationships between social vigilantism and selective exposure, we made no predictions about social vigilantism and selective exposure in the current experiments. Social vigilantism could be negatively related to selective exposure because of a greater tendency to pay attention to (i.e., not ignore) a message that one wants to argue against. Alternatively, social vigilantism could be positively related to selective exposure because having less appreciation of opposing viewpoints and a greater belief in the superiority of one’s own positions may lead those with higher levels of social vigilantism to ignore opposing information. We also included an attitude-*congruent* condition to test whether these patterns of relationships extended to situations where the message people receive is *congruent* with their attitudes about abortion, or whether these patterns of relationships are unique to situations where the message people receive is *in*congruent with their attitudes. Finally, although we were not interested in participants’ possibility of attitude change after watching the ads, which we thought was highly unlikely, we nevertheless included attitude pre- and post-measures as a check.

### 2.1. Methods

#### 2.1.1. Participants

We recruited college students from introductory psychology courses at Kansas State University in exchange for research credit. Our sample (*N* = 232) included 79 men and 153 women, ages 18 to 35 (*M* = 19.60, *SD* = 2.30), most of whom were White (79.9%). All data were collected prior to conducting our analyses.

#### 2.1.2. Materials

To manipulate attitude congruence, we used 2 videos in Experiment 1 (a pro-life ad and a pro-choice ad). The abortion ads, including the arguments presented, were developed specifically for this research. We carried out pilot studies to select arguments based on pro-life and pro-choice participants’ ratings, to ensure that both sets of arguments were rated by their respective supporters as approximately equal in their strength, persuasiveness, valence, and clarity. For more details, see Supplementary material.

##### 2.1.2.1. Abortion ads

We created the pro-life (59 s long) and pro-choice abortion (1 min and 7 s) video ads using matching formats. The ads used intertitles to present the arguments, and had video imagery that by itself would be neutral, but when paired with the arguments would strengthen the arguments being presented. As shown in Table 1, we created intertitles brief enough to be read quickly in short videos, with parallel arguments for each ad.

**Table 1:** Arguments for abortion ads.

	Pro-choice	Pro-life
1	Women today have the right to accomplish anything	Innocent lives should be protected
2	Women of all ages choose to have an abortion	Abortion is irresponsible and unsafe
3	There are many reasons for choosing an abortion	Life begins at conception
4	The rights of the fetus should not outweigh a woman’s rights	Life should be given a chance

The videos share a visual theme that focused on the hands of different people. The pro-Life video^1^ mostly showed the hands of children, doing things like playing with Play-Doh or holding fruit. The pro-choice video^2^ focused on the hands of adult women, doing things like searching on a computer or holding their face. We designed the pro-life ad to show the positives of being a child, and the pro-choice to show the difficulty of deciding to have an abortion.

To immediately inform participants about the position each ad would take, each ad started with an intertitle stating the ad was paid for by either a pro-life or pro-choice group. At the end of each video, a final intertitle told viewers to either “Vote for Choice” (pro-choice video) or to “Choose Life” (pro-life video). The videos had instrumental background music. The pro-choice intertitle texts were slightly longer than the pro-life intertitle texts. The pro-choice video was 8 s longer to give participants time to read the ad’s intertitles.

#### 2.1.3. Procedure and measures

Participants completed all materials online through a Qualtrics survey in the following order: (1) informed consent, (2) demographic information, (3) abortion attitudes pretest, (4) social vigilantism scale, (5) need for cognition scale, (6) the pro-choice or pro-life video ad that was randomly assigned, in a between-groups design, (7) resistance strategies measure, and (8) abortion attitudes posttest. After completing the study, participants were thanked and debriefed.

##### 2.1.3.1. Abortion attitude strength

We measured the strength of participants’ attitudes about abortion using five items modified from Brannon et al. (2007). Participants rated “*The availability of abortion as a legal medical procedure is”* on five 9-point semantic differential scales: good–bad, foolish–wise, unnecessary–necessary, harmful–beneficial, oppose it–favor it. We averaged these items together to create a composite score where higher scores represented more pro-choice attitudes (pretest *M* = 5.17, *SD* = 2.72, α = 0.98; posttest *M* = 5.22, *SD* = 2.78, α = 0.98). Both the pretest and posttest distributions were multimodal, with distinct peaks at one and nine, and a smaller peak near the midpoint of the scale, demonstrating many participants had strong attitudes about abortion.

##### 2.1.3.2. Social vigilantism

We used the social vigilantism scale (Saucier and Webster, 2010) to measure individual differences in the extent to which people generally think their beliefs are superior to others’ beliefs and have a desire to impress their beliefs onto others. Participants responded to the items (e.g., “*I feel as if it is my duty to enlighten other people”*) on 1 (*Strongly Agree*) to 9 (*Strongly Disagree*) scales. We averaged the 14 items to create composite scores where higher values represented higher levels of social vigilantism (*M* = 5.02, *SD* = 1.10, α = 0.85).

##### 2.1.3.3. Need for cognition

We measured people’s preference for, and enjoyment of, deliberate thinking using the need for cognition scale (Cacioppo and Petty, 1982). Participants responded to the items (e.g., “*I would prefer complex to simple problems”*) on 1 (*Strongly Agree*) to 9 (*Strongly Disagree*) scales. We averaged the 18 items (reverse-scoring appropriate items) to create composite scores where higher values represented higher levels of need for cognition (*M* = 5.46, *SD* = 0.92, α = 0.84).

##### 2.1.3.4. Resistance strategies

To measure participants’ intentions to resist persuasion in response to viewing the pro-choice or pro-life video, we used items developed by Jacks and Cameron (2003) and Saucier et al. (2014) to measure eight different resistance strategies (e.g., counterarguing, attitude bolstering). Participants responded to these items with the instructions to “*rate how likely you are to respond in this way to the person who showed you the video*.” While we were only interested in counterarguing and selective exposure, we included the other items as filler material to distract participants from the nature of our study. To reduce the number of variables in our analyses, we averaged the two counterarguing items (e.g., *Respond by thinking about or verbalizing why the person’s arguments are faulty*) to create a composite score (*M* = 4.48, *SD* = 2.05, α = 0.60), and the two selective exposure items (e.g., *Respond by tuning-out the arguments that contradict my position*) to create a composite score (*M* = 3.02, *SD* = 1.87, α = 0.75).

### 2.2. Results

The distribution of abortion attitudes was predominantly bimodal with the vast majority of participants scoring on one end or the other of the scale. Because we were most interested in the behavior of participants with stronger attitudes about abortion, we excluded participants who scored in the middle range of the scale (3.5 to 6.49) on the pretest of abortion attitudes.^3^ For the remaining 153 participants, we coded whether the participants’ abortion attitude was pro-life (scores 1 to 3.49, *n* = 74, 34 viewed the pro-life video and 40 viewed the pro-choice video) or pro-choice (scores 6.5 to 9, *n* = 79, 38 viewed the pro-life video and 41 viewed the pro-choice video). We created a variable to indicate whether the video was congruent (coded 1) or incongruent (coded 0) with participants’ abortion attitudes. The final sample size we analyzed provided us with power > 80% to detect effect sizes > 0.20.

#### 2.2.1. Attitude change

We first tested our *a priori* assumption that participants with strongly held attitudes about abortion would not change their attitudes after viewing the videos. The results of a 2 (pretest/posttest abortion attitude) × 2 (pro-life/pro-choice video) mixed factorial ANOVA with repeated measures on the first factor showed no evidence of attitude change: abortion attitude *F*(1, 151) = 0.65, *p* = 0.42; abortion attitude × video condition interaction *F*(1, 151) = 0.50, *p* = 0.48. Given that we chose participants with the strongest abortion attitudes, the lack of attitude change after watching a single ad was not surprising but instead is consistent with the idea that people with strongly held attitudes would resist persuasion attempts.

#### 2.2.2. Resistance strategies

Next, we examined the bivariate correlations between social vigilantism, need for cognition, counterarguing, and selective exposure. As predicted, social vigilantism was correlated with intentions to engage in counterarguing (*r* = 0.31, *p* < 0.001). Social vigilantism was not significantly correlated with selective exposure (*r* = 0.11, *p* = 0.173). Counter to our hypothesis, need for cognition was unrelated to counterarguing (*r* = 0.01, *p* = 0.868). However, consistent with our hypothesis, need for cognition was negatively related to selective exposure (*r* = −0.23, *p* = 0.004).

We next tested whether these relationships were moderated by viewing an attitude-congruent or attitude-incongruent persuasion attempt. We entered counterarguing and selective exposure as criterion variables in two separate regression models with attitude congruence (step 1), social vigilantism (step 2), and their interaction (step 3) as predictors. In two additional models, we entered attitude congruence, need for cognition, and their interaction as predictors of counterarguing and selective exposure. As expected, the video’s congruence with participants’ abortion attitudes affected their intentions to counterargue, such that participants intended to counterargue the counter-attitudinal message more than the pro-attitudinal message (Congruent: *M* = 4.07, *SD* = 1.90; Incongruent: *M* = 5.35, *SD* = 2.08; *b* = −1.27, 95% confidence interval lower = −1.91, upper = −0.64, *p* < 0.001). Consistent with the social vigilantism hypothesis, social vigilantism was positively related to counterarguing (*b* = 0.58, 95% confidence interval lower = 0.30, upper = 0.87, *p* < 0.001), and this relationship was not significantly moderated by attitude congruence as indicated by a non-significant social vigilantism X Congruence interaction (*b* = −0.31, 95% confidence interval lower = −0.88, upper = 0.26, *p* = 0.282). Need for cognition was unrelated to counterarguing intentions (*b* = −0.04, 95% confidence interval lower = −0.39, upper = 0.30, *p* = 0.802) and did not interact with the video condition (*b* = 0.04, 95% confidence interval lower = −0.65, upper = 0.74, *p* = 0.899).

Surprisingly, selective exposure intentions did not significantly differ by condition (Congruent: *M* = 2.88, *SD* = 1.88; Incongruent: *M* = 3.05, *SD* = 1.73; *b* = −0.17, 95% confidence interval lower = −0.75, upper = 0.41, *p* = 0.559). Consistent with the need for cognition hypothesis, need for cognition was negatively related to selective exposure (*b* = −0.46, 95% confidence interval lower = −0.76, upper = −0.16, *p* = 0.003) and did not interact with attitude congruence (*b* = 0.23, 95% confidence interval lower = −0.38, upper = 0.84, *p* = 0.462). Social vigilantism was not significantly related to selective exposure (*b* = 0.18, 95% confidence interval lower = −0.09, upper = 0.45, *p* = 0.180) and, interestingly, did not interact with the attitude congruence of the video (*b* = 0.11, 95% confidence interval lower = −0.44, upper = 0.65, *p* = 0.698).

### 2.3. Discussion

Our findings were consistent with previous research showing a persuasion attempt incongruent with a strongly held attitude is unlikely to change that attitude and would likely elicit stronger intentions to counterargue than a persuasion attempt congruent with that attitude. We found support for our social vigilantism hypothesis—higher levels of social vigilantism were related to stronger intentions to counterargue. However, social vigilantism did not interact with the effects of the attitude congruence of the persuasive message. Rather, our data suggest people’s tendencies to argue and impress their beliefs on others may be an omnipresent goal, regardless of whether a message agrees or disagrees with their position on the issue. Whether social vigilantism is related to intentions to ignore attitude-relevant information was inconclusive.

Furthermore, we found support for the hypothesis that people with greater need for cognition would be less likely to ignore attitude-incongruent information. However, need for cognition was not correlated with counterarguing, suggesting that while need for cognition may be related to attending to, rather than ignoring, the information in a persuasive message, such attention may not be for the purpose of counterarguing.

Overall, these data suggest individual differences in social vigilantism and need for cognition are important for understanding how people process attitude-relevant information. The chronic motivation to influence others’ attitudes by people high in social vigilantism appears to be related to their intentions to counterargue, regardless of the attitude-consistency of the topic. Furthermore, dispositional tendencies for careful thought are also related to being less likely to ignore information relevant to a strongly held attitude.

## 3. Experiment 2

In Experiment 1, social vigilantism was related to counterarguing regardless of whether the persuasive message was congruent or incongruent with participants’ attitudes about abortion. Similarly the attitude congruence of the persuasive message did not moderate the negative relationship between need for cognition and selective exposure. In Experiment 2, we assessed how social vigilantism and need for cognition relate to resistance strategies in the context of an *un*controversial message to test whether social vigilantism and need for cognition predict counterarguing and selective exposure (respectively) more generally, regardless of the attitude-relevance of the information in a persuasive message. In a between-groups design, we used the same pro-choice and pro-life videos but added a third condition in which participants viewed a short video containing a relatively less politically controversial message about disabilities. This allowed us to test whether the associations between social vigilantism and counterarguing or need for cognition and selective exposure are specific to attitude-relevant persuasion attempts or whether they generalize to participants’ responses to a message less relevant to attitudes about abortion. Additionally, in Experiment 1, we measured abortion attitudes at the start and end of the study to test our assumption that strongly held attitudes would not change. However, the pre-measure of abortion attitudes may have primed participants’ attitudes about abortion, and thereby may have affected how they responded to the items measuring intentions to counterargue or ignore the persuasion attempt. Therefore, in Experiment 2 we waited to measure participants’ attitudes about abortion until the end of the study and did not test for attitude change because the results of Study 1 strongly supported our *a priori* assumption that participants’ attitudes about abortion were very *un*likely to change after exposure to either of our single political advertisements.

### 3.1. Methods

#### 3.1.1. Participants

We recruited a new sample of college students from introductory psychology courses at Kansas State University in exchange for research credit. Our sample (*N* = 234) included 102 men and 132 women, ages 18 to 28 (*M* = 19.18, *SD* = 1.72), most of whom were White (81.4%). All data were collected prior to conducting our analyses.

#### 3.1.2. Procedure and measures

We used the same procedure as Experiment 1, with the exception that participants only completed the abortion attitudes items at the end of the study. We also included a Non-controversial video^4^ condition in the form of a public service announcement with the concluding message text: *Just because you do something differently, does not mean you are “disabled.”* Visually, the ad is set on a series of steps, and people go up and down them in different and creative ways (e.g., dancing and crab walking). The pro-choice and pro-life videos were the same as Experiment 1. Participants were randomly assigned one of the three videos. Composite variables for each of the measures were calculated as described in Experiment 1 (abortion attitudes *M* = 5.24, *SD* = 2.80, α = 0.99; social vigilantism *M* = 5.18, *SD* = 1.09, α = 0.85; need for cognition *M* = 5.83, *SD* = 1.05, α = 0.84; counterarguing *M* = 4.40, *SD* = 1.99, α = 0.63; selective exposure *M* = 3.12, *SD* = 1.91, α = 0.81). Again, the distribution of abortion attitudes was multimodal, with distinct peaks at one and nine, and a smaller peak near the midpoint of the scale.

### 3.2. Results

As in Experiment 1, we excluded participants who scored in the middle range of the scale (3.5 to 6.49) on abortion attitudes, resulting in a sample of 160 participants. We coded participants’ abortion attitudes as described in Study 1 (pro-life *n* = 72, 24 viewed the non-controversial video, 28 viewed the pro-life video, and 20 viewed the pro-choice video; pro-choice *n* = 88, 29 viewed the non-controversial video, 28 viewed the pro-life video, and 31 viewed the pro-choice video). The final sample size we analyzed provided us with power > 80% to detect effect sizes > 0.20.

#### 3.2.1. Resistance strategies

We first examined the bivariate correlations between social vigilantism, need for cognition, counterarguing, and selective exposure. As predicted, social vigilantism was once again correlated with intentions to engage in counterarguing (*r* = 0.32, *p* < 0.001). However, in contrast to Experiment 1, social vigilantism was significantly positively correlated with selective exposure (*r* = 0.21, *p* = 0.008). Need for cognition was again unrelated to counterarguing (*r* = 0.07, *p* = 0.366). As expected, need for cognition was again negatively related to selective exposure (*r* = −0.20, *p* = 0.013).

We next tested whether these relationships were moderated by attitude congruence. We entered counterarguing and selective exposure in separate regression models with the abortion attitude congruence of the video entered as dummy-coded aspects of the three video conditions (Congruent, Incongruent, or Neutral for the uncontroversial video in step 1), social vigilantism (step 2), and their interactions (step 3) as predictors. In additional models, we entered attitude congruence, need for cognition, and their interactions as predictors.

We replicated the finding from Experiment 1 that the attitude congruence of the persuasion attempt affected intentions to counterargue (Congruent: *M* = 3.83, *SD* = 2.18; Incongruent: *M* = 5.16, *SD* = 1.96; Congruent—Incongruent *b* = −1.33, 95% confidence interval lower = −2.11, upper = −0.54, *p* < 0.001). The Neutral condition (*M* = 4.45, *SD* = 2.00) did not differ from either the Congruent (Neutral—Congruent *b* = 0.62, 95% confidence interval lower = −0.16, upper = 1.40, *p* = 0.116) or Incongruent (Neutral—Incongruent *b* = −0.71, 95% confidence interval lower = −1.50, upper = 0.09, *p* = 0.082) conditions. Replicating the results from Experiment 1, we found social vigilantism was positively related to counterarguing (*b* = 0.63, 95% confidence interval lower = 0.37, upper = 0.88, *p* < 0.001), and social vigilantism did not interact with attitude congruence (*p*s > 0.529). Again, we found need for cognition was unrelated to counterarguing (*b* = 0.19, 95% confidence interval lower = −0.11, upper = 0.49, *p* = 0.207) and did not interact with attitude congruence (*p*s > 0.120).

For selective exposure, as expected, we found intentions to ignore the persuasion attempt were highest in the Incongruent condition (*M* = 3.74, *SD* = 2.09; Congruent—Incongruent *b* = −1.37, 95% confidence interval lower = −2.09, upper = −0.65, *p* < 0.001; Neutral—Incongruent *b* = −0.80, 95% confidence interval lower = −1.53, upper = −0.07, *p* = 0.032), followed by the Neutral condition (*M* = 2.93, *SD* = 1.99; Neutral—Congruent *b* = 0.57, 95% confidence interval lower = −0.15, upper = 1.28, *p* = 0.119), and lowest the Congruent condition (*M* = 2.37, *SD* = 1.58). In contrast to Experiment 1, social vigilantism was positively related to selective exposure (*b* = 0.40, 95% confidence interval lower = 0.15, upper = 0.65, *p* = 0.002). However, social vigilantism did not interact with attitude congruence (*p*s > 0.307). Replicating results from Experiment 1, we found need for cognition was negatively related to selective exposure (*b* = −0.31, 95% confidence interval lower = −0.58, upper −0.03, *p* = 0.027) and the interactions between need for cognition and attitude congruence were non-significant (*p*s > 0.088).

### 3.3. Discussion

In Experiment 2, we replicated our previous findings supporting our social vigilantism hypothesis: once again, higher levels of social vigilantism were found to be related to stronger intentions to counterargue. We additionally replicated the finding that the relationship between social vigilantism and counterarguing was not moderated by the attitude congruence of the persuasive message. Replicating this finding gives us stronger confidence in concluding people who perceive their beliefs are superior and try to impress them onto others, may feel a greater need to argue in response to persuasion attempts—regardless of whether that attempt is congruent or incongruent with a strongly-held attitude.

Although social vigilantism did not correlate with intentions to ignore attitude-relevant messages in Experiment 1, we found a positive relationship between social vigilantism and selective exposure in the current experiment. The only difference between the two experiments was the inclusion of a less controversial message. It is possible that social vigilantism could be related to ignoring persuasive messaging about uncontroversial issues and including the neutral video increased the correlation between social vigilantism and selective exposure. However, prior research has shown that social vigilantism is related to more active resistance strategies (e.g., counterarguing, impressing one’s own views on others) and unrelated to selective exposure regardless of the importance of the issue (Saucier et al., 2014, Study 2) suggesting that social vigilantism should be unrelated to selective exposure even for less controversial issues. We provided a further test of the relationship between social vigilantism and selective exposure in Experiment 3.

## 4. Experiment 3

Experiment 3 tested if the self-reported counterarguing and selective exposure intentions in Experiments 1 and 2 are related to participants’ subsequent memory performance. Based on the resistance strategy results, people high in social vigilantism should be more engaged with all material, which would likely result in better memory for all video material. Because social vigilantism was inconsistently related to self-reported selective exposure across Experiments 1 and 2, memory for the contents of a persuasive message may provide a better test of selective exposure behavior. If social vigilantism is related to memory for attitude-relevant information, then it would suggest that social vigilantism is at best unrelated, if not negatively related, to selective exposure. Furthermore, in Experiments 1 and 2, people with higher levels of need for cognition showed less selective exposure for attitude-congruent and -incongruent information, so they may as a result have better memory for attitude-relevant information.

Importantly, the behaviors involved in selective exposure and counterarguing are inherently related to attention (i.e., selective exposure assumes fewer attentional resources are used to process counter-attitudinal information). As such, it is important to consider the effect the stimuli used may have on attention. Recent work on visual attention to videos shows that highly produced films and advertisements create a phenomenon known as attentional synchrony (Dorr et al., 2010; Smith and Mital, 2013), in which people show high convergence in where they look in videos on a moment-to-moment basis. Further, attentional synchrony persists despite large differences in top-down processes, which has been termed *the tyranny of film* (Loschky et al., 2015; Hutson et al., 2017). Given that memory is highly correlated with what a person attends to Loftus (1972), Hollingworth and Henderson (2002), Tatler et al. (2005), Zelinsky and Loschky (2005), and Pertzov et al. (2009), if the ads guide attention despite differences in attitude congruence, there could be a dissociation between participants reported resistance strategies from Experiments 1 and 2, and their memory. In other words, participants may believe they are engaging with the content differently due to their beliefs, but the video composition may be involuntarily guiding their attention and controlling what they recall.

### 4.1. Memory experiment hypotheses

#### 4.1.1. Selective exposure

The selective exposure hypothesis predicts participants will have better memory for attitude-congruent vs. attitude-incongruent information.

#### 4.1.2. Social vigilantism

Based on the results from Experiments 1 and 2, the social vigilantism hypothesis predicts that because social vigilantism was positively correlated with intentions to counterargue, participants higher in social vigilantism will be more likely to engage in processing attitude-congruent and attitude-incongruent information. This would result in better memory for both abortion ads but show no relationship for the less controversial video. Alternatively, if social vigilantism is positively related to selective exposure, participants higher in social vigilantism should show worse memory for the information in the attitude-incongruent videos because selective exposure is a resistance strategy that involves ignoring information that is inconsistent with one’s attitude.

#### 4.1.3. Need for cognition

Based on the results from Experiments 1 and 2, the need for cognition hypothesis predicts that because need for cognition was negatively related to selective exposure, participants higher in need for cognition will attend more to the information, and thus have better memory for the content of *all* of the videos.

#### 4.1.4. Tyranny of film (null)

The tyranny of film hypothesis predicts that, due to the control filmmakers have over what information is presented, participants will have similar memory for the ads regardless of their attitudes, social vigilantism, or need for cognition.

### 4.2. Methods

#### 4.2.1. Participants

A new sample of 118 participants were recruited from introductory psychology courses at Kansas State University to participate in the experiment (ages 18–41 [*M* = 19.8, *SD* = 3.1], 57% female, 86% were White). The obtained sample size provided >80% power to detect effect sizes > 0.10. All data were collected prior to conducting our analyses.

##### 4.2.1.1. Individual difference scores

Participants completed the same individual difference measures for the memory experiment as in Experiments 1 and 2 (abortion attitudes *M* = 4.89, *SD* = 2.82; social vigilantism *M* = 4.98, *SD* = 1.22; need for cognition *M* = 5.28, *SD* = 1.06). Most participants identified as being either strongly pro-life or pro-choice, with a smaller group of participants indicating they had no strong attitude one way or the other. There were roughly an equal number of participants who identified as pro-life and pro-choice. In the current experiment, we elected to include the complete range of abortion attitude scores to use the full power of our sample size.

### 4.3. Stimuli

#### 4.3.1. Videos

Participants viewed the same abortion ads as in Experiments 1 and 2 and the same non-controversial ad as in Experiment 2.

#### 4.3.2. Memory test items

The memory test stimuli were developed to measure both recall and recognition memory, and visual and verbal memory. Free recall memory was of interest, because previous work has shown it may be more susceptible to top-down effects than recognition memory (Mandler, 2008). For free recall memory, participants were given prompts to recall as much verbal and then visual information as possible, as if they were explaining the video to a friend who had not seen it.

Participants completed 3 types of recognition memory items: argument recognition, visual multiple choice, and visual recognition. Argument recognition memory items presented participants with an argument, and they indicated whether it was worded exactly as in the ad they saw, or if it was reworded in some way (e.g., synonyms were used and/or verb tense was changed). Visual multiple-choice questions had a stem asking about a visual element of an ad (e.g., “What fruit was shown in the ad?”). Each question had four answer options. Finally, Visual Recognition memory items used video stills taken from the ad. For these items, participants indicated whether the image was presented as it originally was in the ad, or if it was mirror reversed (i.e., left/right reversed). All items were scored as correct (1) or incorrect (0).

Importantly, we used memory measures for different modalities and different levels of representation (Van Dijk and Kintsch, 1983), because memory effects could be different based on the how the viewer interacts with an ad. For example, if a viewer engages in counterarguing, they may be more likely to counterargue the text information presented, rather than the images. As such, the influence of counterarguing could be specific to memory for the text.

### 4.4. Procedure

The current experiment was conducted online via Qualtrics. Participants first watched all three ads (with order randomized for each participant) so that we could compare memory for each ad within-participants. After the videos, participants responded to the memory questions. The questions were organized into blocks based on their type in the following order for all participants: free recall, argument recognition, visual multiple choice, and visual recognition. Free recall questions were presented before argument recognition questions so that the presentation of the recognition items did not influence responses to the free recalls. Similarly, visual multiple-choice questions were presented before the visual recognition memory items so that seeing the visual recognition items could not influence responses to the visual multiple-choice questions.

### 4.5. Analyses

We ran multilevel logistic regressions separately for each video type (non-controversial & abortion ads), as well as for the different types of memory items. The random effects structure for all analyses included the participant and memory item. This random effects structure was determined to be best based on AIC values when compared to a random effects structure that only included the participant (Burnham and Anderson, 2004). To determine the best fixed effects (predictor) structure most likely to generalize at the population level, we used a model testing procedure, and selected the best model using AIC values to reduce the likelihood of Type I errors.

#### 4.5.1. Signal detection analyses

The Argument and Visual Recognition memory items used an Old (i.e., seen in the experiment video)/New (i.e., not seen in the experiment video) format, which allowed us to use signal detection analyses. We conducted the signal detection analyses using logistic multilevel models with the probit link function (DeCarlo, 1998; Wright et al., 2009).

There are a few keys to interpreting signal detection analyses done with multilevel models. First, the intercept of the model is the overall bias (i.e., c = decision *criterion,* do participants disproportionately respond “Old” or “New”?). Second, the first predictor in the model is always whether the memory item was an “Old” or “New” item, and this is the overall sensitivity (i.e., d', the ability to discriminate between “Old” and “New” items). Effects of predictors on bias are indicated by adjustments to the intercept. Effects of the predictors on sensitivity are indicated by their interaction with “Old/New.”

#### 4.5.2. Free recall scoring

Unfortunately, participant free recall responses were very short (*M* = 19.4 words; *SD* = 15.2 words), and some participants simply wrote that they did not remember the video. This resulted in a floor effect, and there were no clear effects or trends with any of the predictor variables. As such the free recall data is not presented here.

### 4.6. Results

#### 4.6.1. Results overview

Overall, Experiment 3 showed partial support for the tyranny of film and social vigilantism hypotheses, as well as some general top-down effects independent of attitude congruence that were not hypothesized *a priori*. Interestingly, many of these effects were found across measures and videos. Also, in contrast to Experiments 1 and 2, we found no effects of need for cognition on any of the memory measures, thus providing no support for the need for cognition hypothesis in terms of memory.

### 4.7. Non-controversial ad

#### 4.7.1. Argument recognition memory

The best model only included attitude and social vigilantism as predictors (i.e., the simplest model). As expected, given the non-controversial nature of the ad, neither predictor influenced sensitivity or bias (all *p*’s > 0.05). In other words, the model indicated individual differences did not significantly predict argument recognition memory for the non-controversial ad. Overall, for the non-controversial video argument recognition items, participants had very low sensitivity (*d* ' = 0.16, *z* = 0.90, *p* = 0.368), but they showed a strong “Old” bias (c = 0.62, *z* = 6.99, *p* < 0.001). Thus, participants did well for unchanged “Old” items, but were well below chance for reworded “New” memory items.

#### 4.7.2. Visual recognition memory

**Figure 1:**
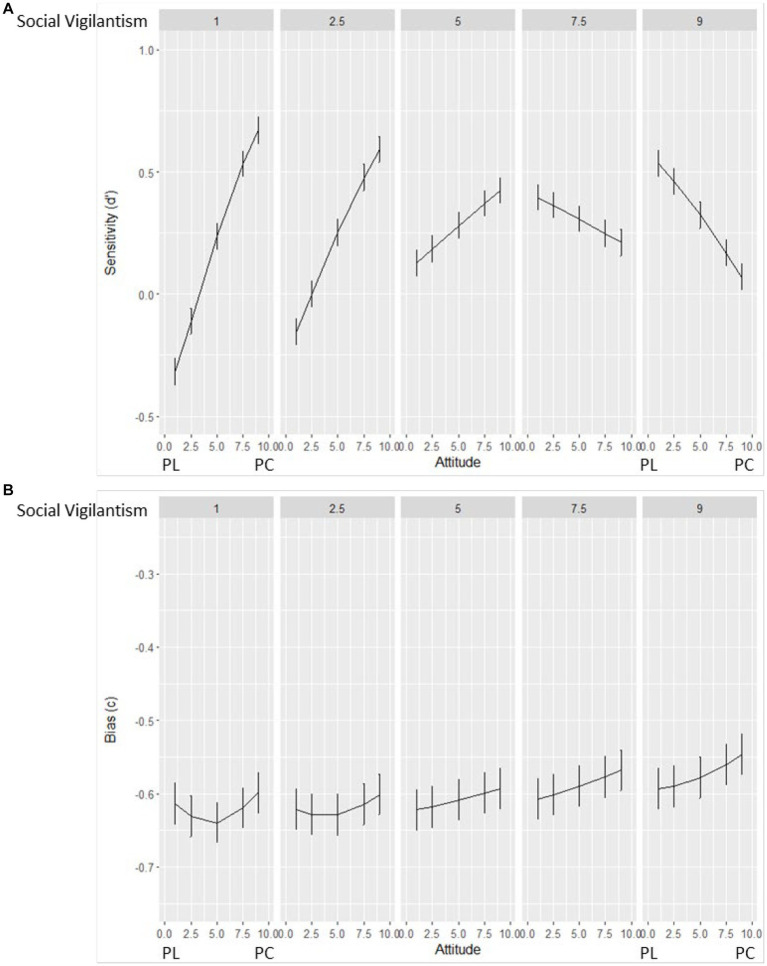
Signal detection analysis for Non-controversial visual recognition memory. **(A)** The Y-axis is the predicted sensitivity (d’) to the visual recognition memory items. The X-axis is attitude PL = pro-life, PC = pro-choice (1 = most pro-life; 9 = most pro-choice). The panels labeled at the top of the graph are cross sections of the social vigilantism (SV) measure (1 = Very low in SV; 9 = Very high in SV). **(B)** The Y-axis is the predicted bias (c). All other axes are the same as for **(A)** (Attitude on the X-axis and Social vigilantism for the panels). Error bars are 1 standard error.

Surprisingly, given the non-controversial nature of the ad, for visual recognition memory the best model included the interaction of attitude and social vigilantism with recognition memory item type (“Old” vs. “Mirror reversed”). As shown in Table 2, participants were sensitive to the visual recognition memory items, and pro-choice participants showed higher overall sensitivity. Interestingly, however, the interaction of attitude and social vigilantism influenced sensitivity. As shown in Figure 1A, the interaction between attitude and social vigilantism on sensitivity created an “arch” shape. Specifically, for people higher in social vigilantism, pro-life participants were more sensitive than those who were pro-choice; conversely, for people lower in social vigilantism, this relationship reversed—pro-choice participants were more sensitive than pro-life participants (Figure 1A). Surprisingly, these results show, even when a video is on a *non*-controversial topic, a person’s attitude toward a controversial topic and their level of social vigilantism, can interact to influence their visual recognition memory. None of the independent variables significantly influenced bias.

**Table 2:** Summary of multilevel logistic signal detection analysis for non-controversial ad visual recognition memory.

Variable	*B*	*SE(B)*	*z*	Sig. (*p*)
Intercept [Bias]	0.29	0.17	1.79	0.072
“Old”/“New” [sensitivity]	0.73	0.33	2.23	0.026
Attitude	−0.005	0.02	−0.25	0.805
Social vigilantism	−0.02	0.04	−0.43	0.664
“Old”/“New” × attitude	0.10	0.04	2.80	0.005
“Old”/“New” × SV	0.03	0.09	0.38	0.703
Attitude × SV	−0.005	0.01	−0.37	0.713
“Old”/“New” × attitude × SV	−0.07	0.03	−2.51	0.012

The intercept of the model is the overall bias. “Old”/“New” shows the overall sensitivity to the memory items. Attitude, Social Vigilantism (SV), and Attitude × SV show adjustments to bias. Interactions with “Old”/“New” show adjustments to sensitivity. The continuous variables were centered for the interaction. Statistically significant effects are shaded.

#### 4.7.3. Visual multiple choice

The three individual difference variables in the accuracy model showed no significant effects on participants’ memory for visual details (*p*’s > 0.05). The performance predicted by the model was relatively low, 38%, but significantly above chance performance (25%).

When taken together, the recognition memory item results for the non-controversial ad were mostly consistent with our expectation that differences in abortion attitudes and social vigilantism would not have effects. However, there was one exception. For visual recognition memory, there were effects of attitude, social vigilantism, and their interaction.

### 4.8. Abortion ads

#### 4.8.1. Argument recognition memory

As with the non-controversial ad, there was only a significant “Old” bias. Participants were more likely to indicate that memory items had appeared in the video (c = −0.61, *z* = 5.39, *p* < 0.001). Although none of the individual difference measures were significant, there was a non-significant trend toward an interaction of attitude congruence and social vigilantism on sensitivity (*b* = −0.04, *z* = −1.76, *p* = 0.08). Participants lower in social vigilantism tended to show higher sensitivity for the attitude-congruent video, but participants higher in social vigilantism tended to show better memory for the attitude-incongruent video. In contrast to Experiments 1 and 2 where social vigilantism was related to counterarguing regardless of the attitude congruence of the persuasive message, our memory results suggest that social vigilantism was positively related to engaging with attitude-incongruent messages and negatively related to engaging with attitude-congruent messages.

#### 4.8.2. Visual recognition memory

For the abortion ads, we replicated some of the attitude and social vigilantism effects found for the non-controversial ad. The best model included “Old”/“New,” Attitude, and social vigilantism, but there were no attitude congruence effects. Overall, as shown in Table 3, there was an “Old” bias, and participants were sensitive to the memory items. Interestingly, participants who were more pro-choice had higher sensitivity. This effect was not predicted.

**Table 3:** Summary of multilevel logistic signal detection for abortion ad visual recognition memory.

Variable	*B*	*SE(B)*	*t*	Sig. (*p*)
Intercept [Bias]	−0.46	0.08	5.60	<0.001
“Old”/“New” [sensitivity]	0.79	0.16	4.80	<0.001
Attitude	0.0004	0.009	−0.04	0.97
Social vigilantism	0.03	0.02	−1.45	0.15
“Old”/“New” × attitude	0.05	0.02	2.58	0.01
“Old”/“New” × SV	−0.06	0.05	−1.22	0.22
Attitude × SV	−0.01	0.007	1.41	0.16
“Old”/“New” × attitude × SV	−0.01	0.01	−1.07	0.28

The intercept of the model is the overall bias. “Old”/“New” shows the overall sensitivity to the memory items. Attitude, Social vigilantism (SV), and Attitude × SV show adjustments to bias. Interactions with “Old”/“New” show adjustments to sensitivity. The continuous variables were centered for the interaction. Statistically significant effects are shaded.

#### 4.8.3. Visual multiple choice

**Figure 2:**
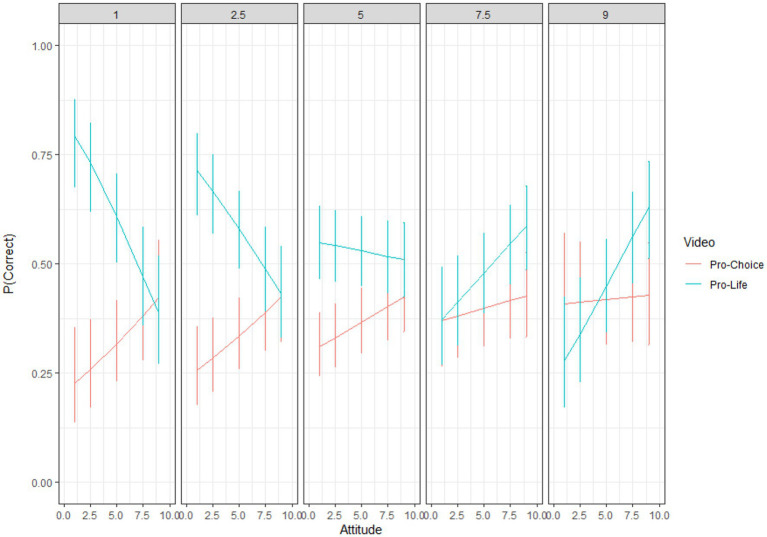
Abortion ads’ visual multiple choice. Y-axis is predicted accuracy. The X-axis shows abortion attitude (low scores = more pro-life; high scores = more pro-choice). The panels labeled at the top of the graph are cross sections of the social vigilantism (SV) measure (1 = very low in SV; 9 = very high in SV). Error bars are 1 standard error.

Consistent with the hypothesis that social vigilantism would be related to better memory, higher levels of social vigilantism related to better memory for attitude-*in*congruent content (Table 4; Figure 2). This U-shaped pattern is especially clear for the pro-life video. At lower levels of social vigilantism, pro-life participants showed better memory, and, at higher levels of social vigilantism, pro-choice participants showed better memory. For the pro-choice video, we found approximately the same general trend in reverse, a partial “arch” pattern, similar to visual recognition memory for the non-controversial ad (Figure 1A), and the non-significant trend we found for argument recognition memory. In this case, at lower levels of social vigilantism, pro-choice participants had better memory for the pro-choice video; however, the slope did not reverse direction at higher levels of social vigilantism.

**Table 4:** Summary of multilevel logistic for abortion ad visual multiple-choice memory.

Variable	*B*	*SE(B)*	*z*	Sig. (*p*)
Intercept	−0.26	0.23	−0.94	0.349
Attitude	0.02	0.02	0.90	0.367
Social vigilantism	−0.01	0.06	−0.23	0.816
Video	−0.33	0.23	−1.47	0.141
Att. × SV	0.02	0.02	1.10	0.271
Att. × video	0.04	0.02	2.20	0.028
Video × SV	0.07	0.05	1.5	0.134
Att. × SV × video	−0.03	0.01	−2.33	0.020

Describes model for predicted accuracy for the visual multiple-choice memory questions. The continuous variables were centered for the interaction. Statistically significant effects are shaded. SV = social vigilantism.

A trend that emerged from the abortion ad memory results was that social vigilantism moderated the effect of attitude congruence on memory performance. People lower in social vigilantism had better memory for attitude-congruent items, while those higher in social vigilantism had better memory for attitude-*in*congruent items—which produces a “U” pattern, or conversely an arch pattern. Although this effect was only significant for the visual multiple-choice questions, the argument recognition items trended in the same direction. It is interesting that social vigilantism moderated the effects of attitude congruence for items based on visual information and trended for argument items, but not for the visual recognition items. One possibility, based on Mandler’s (2008) dual process model of recognition memory, is immediate visual recognition memory operates at a perceptual level (Langley et al., 2008) that may not be affected by top-down (here, attitude or personality-driven) processing.

### 4.9. Discussion

Both attitude congruence and social vigilantism influenced memory for political ads, but the presence of effects varied with memory type (argument recognition vs. visual multiple choice). First, for the non-controversial ad, which was included as a baseline condition, viewers’ visual recognition memory surprisingly showed differences based on their attitudes and level of social vigilantism. Participants who were pro-choice were more sensitive for visual recognition memory items (which was also found for the abortion ads). Thus, the non-controversial ad showed top-down effects of attitude and social vigilantism on memory, even for a non-controversial topic. Additionally, the arch shaped interaction created in Figure 1A was also partially found in Figure 2 for the abortion ads visual multiple-choice memory measure.

Importantly, the abortion ad results showed effects of both attitude and social vigilantism. At higher levels of social vigilantism, participants showed better memory for attitude-*in*congruent information. This effect was strongest for the visual multiple-choice measure, and somewhat less so for argument recognition. These findings combine to indicate an effect of attitude congruence, social vigilantism, and their interaction on memory for politically controversial content. However, in contrast to Experiments 1 and 2, we found no support for the need for cognition hypothesis.

An interesting insight from the current work and an area for future inquiry is the level of memory representation at which the top-down effects occurred. Van Dijk and Kintsch (1983) proposed three levels of representation for text: surface, propositional, and situational. Subsequent work verified people do encode and retrieve all three levels of representation (Schmalhofer and Glavanov, 1986; Fletcher and Chrysler, 1990). In the current work, the recognition memory items manipulated the surface structure of the arguments presented in the videos, but the propositional representations of the text base remained the same (i.e., the wording of the arguments changed, but the meaning stayed the same). Thus, the recognition memory results showed individual difference effects at the surface level.

## 5. General discussion

We live in a highly politically divisive era, in which political discourse is increasingly insular. People are continuously exposed to political video content through various media, which is increasingly filtered to create political “echo chambers” consistent with people’s pre-existing beliefs. Highly produced videos, such as political ads, are designed to create similar attentional patterns across viewers, which could produce similar memory across viewers as well. Some people, who are high in social vigilantism, choose to actively debate with those with whom they disagree, often trying to persuade them to change their minds, while others, who are high in need for cognition, may be less argumentative, but still greatly value thinking through difficult problems. Such individual differences could create large differences in people’s memory for the contents of political videos. This inspires our key question: how do individual differences in social vigilantism and need for cognition interact with the attitude congruence of political videos in determining viewers’ reported resistance strategies and what they remembered from such videos?

We tested several hypotheses about how social vigilantism and need for cognition would interact with responses to political content that was congruent vs. incongruent with viewers’ attitudes. The Tyranny of Film hypothesis predicted individual differences in social vigilantism and need for cognition would not be predictive of viewers’ memory, because video makers are experts at guiding viewers’ attention and the information available to process. The three alternative competing hypotheses stated people may either (i) avoid or tune-out counter-attitudinal information (selective exposure), or (ii) engage with incongruent information more if they had higher belief superiority and desire to impress their beliefs on others (social vigilantism), or (iii) that viewers’ who enjoy engaging in demanding cognitive tasks would engage more (need for cognition).

In Experiments 1 and 2, we found evidence of both need for cognition and social vigilantism affecting the resistance strategies adopted when viewing videos containing controversial political content congruent vs. incongruent with their attitudes. As expected, people reported greater intentions to resist persuasive video content that was attitude-incongruent in terms of counterarguing and selective exposure. However, consistent with the social vigilantism hypothesis, those higher in social vigilantism were more likely to report the intention to counterargue than those who were lower in social vigilantism. Interestingly, people higher in social vigilantism were more likely to report intentions to counterargue even when the video content was attitude-congruent. We also found evidence for the need for cognition hypothesis, with people higher in need for cognition reporting lesser intent to practice selective exposure to ads inconsistent with their attitudes.

In Experiment 3, we investigated whether these reported intentions were consistent with viewers’ memory for the same political videos and found those higher in social vigilantism had better recognition memory for ad content that was attitude-incongruent. Notably, for people lower in social vigilantism these results showed evidence consistent with selective exposure, though in Experiment 1, participants did not report intending to ignore attitude-incongruent political video content, and in Experiment 2, attitude-congruence did not moderate the significant positive relationship between social vigilantism and intention to engage in selective exposure. Thus, the data across Experiments 1–3 show a degree of divergence between our self-report measures and memory for information one disagrees with. Findings from Experiment 3 are also consistent with the hypothesis that higher levels of social vigilantism would be associated with counterarguing resistance strategies, because the better memory for attitude-incongruent content suggests people higher in social vigilantism might be motivated to gather information about opposing viewpoints in order to directly challenge those arguments. Experiment 3 found no support for the need for cognition hypothesis. Nevertheless, Experiments 1 and 2 showed that participants higher in need for cognition were less likely to report intentions to engage in selective exposure for attitude-incongruent information. Future work should address this inconsistency.

Interestingly, we also found effects of attitude, but not attitude congruence, and social vigilantism, for memory for the non-controversial ad. This ad was included as a baseline measure, for which we expected to find no attitude effects. Nevertheless, viewers’ (seemingly) irrelevant attitudes toward a different and highly controversial political topic (abortion) produced differences in their memory for the *non*-controversial ad. The same pattern of results (arch or “U” shape) was also partially found for the visual multiple choice measure for the abortion ads. Future work will test whether this is a reliable relationship, which may be similar in nature to the effects of political ideology on attention (Dodd et al., 2012).

### 5.1. Limitations and future directions

People do not always behave in ways consistent with how they say they will behave. Thus, one limitation of Experiments 1 and 2 is that participants self-reported their intentions to engage in counterarguing and selective exposure. It may be socially undesirable for people to claim they would ignore or counterargue against attitude-incongruent persuasion attempts, and this may have affected how participants self-reported their intentions to engage in resistance strategies. Additionally, in Experiments 1 and 2, higher levels of social vigilantism were related to stronger intentions to counterargue regardless of whether the video was congruent or incongruent with participants’ attitudes. This finding might have been due to participants attempting to maintain consistent responses across the counterarguing measures and the social vigilantism items pertaining to argumentative tendencies. However, our memory measures in Experiment 3, which did not have these same limitations, provided further support for the social vigilantism hypothesis. To be more confident in concluding higher levels of social vigilantism are related to counterarguing against attitude-incongruent positions, future research could also assess whether higher levels of social vigilantism are associated with paying more attention to attitude-incongruent information. For example, eye movement or eye blink measures (e.g., Nakano et al., 2009; Andreu-Sánchez et al., 2021a,b) would be more direct measures of attention.

Additionally, because the social vigilantism scale seems to capture both belief superiority as well as motivations to impress those beliefs onto others, it would be interesting to examine which of these two constructs is more strongly associated with counterarguing behaviors. This could be studied by including both the social vigilantism scale and the general belief superiority scale (Raimi and Jongman-Sereno, 2020) as competing predictors of counterarguing behavior. It may be that belief superiority alone would be sufficient for eliciting several of the resistance to persuasion strategies identified in past research, but we suspect that the motivation to impress one’s beliefs onto others would more strongly predict counterarguing specifically.

### 5.2. Conclusion

Our research contributes to our understanding of how people resist politically charged attempts at persuasion by showing how higher levels of social vigilantism and need for cognition are related to greater intentions to engage with, rather than tune out, information that opposes their strongly held attitudes. The results of our memory study further demonstrate how levels of social vigilantism relate to cognitive processes that may facilitate engagement through better memory for opposing arguments, perhaps because individuals higher in social vigilantism are tracking the information they are motivated to argue against. Together, our findings highlight the significant role individual differences play in how people process and respond to attempts to change their strongly held attitudes.

## Data availability statement

The datasets presented in this study can be found in online repositories. The names of the repository/repositories and accession number(s) can be found at: https://osf.io/k6j3z/?view_only=ed561b0a2a7b4f9ab5ece40c64489211.

## Ethics statement

The studies involving human participants were reviewed and approved by Kansas State University Institutional Review Board. The patients/participants provided their written informed consent to participate in this study.

## Author contributions

SM: manuscript writing and experimental design, materials, data collection, and analyses for Experiments 1 and 2. JH: manuscript writing and experimental design, materials, data collection, and analyses for Experiment 3. MS: manuscript writing and study design, materials, data collection, and analyses for the pilot study. TS, LL, and DS: manuscript writing and advised on the design for all three experiments. MP: creation of video stimuli used in all three experiments. All authors contributed to the article and approved the submitted version.

## Conflict of interest

The authors declare that the research was conducted in the absence of any commercial or financial relationships that could be construed as a potential conflict of interest.

## Publisher’s note

All claims expressed in this article are solely those of the authors and do not necessarily represent those of their affiliated organizations, or those of the publisher, the editors and the reviewers. Any product that may be evaluated in this article, or claim that may be made by its manufacturer, is not guaranteed or endorsed by the publisher.

## Footnotes

^1^ https://www.youtube.com/watch?v=eXKjmc4Ai9A

^2^ https://www.youtube.com/watch?v=qTQXd2FCh_w

^3^On an ordinal scale of 1–9, we would have removed 4–6. However, because the scores were averaged, they were rational numbers rather than whole numbers. We therefore removed participants having the range of values between those that would round up to 4 and those that would round down to 6. Similar results were obtained in our analyses when less conservative selection criteria were used to exclude only participants who scored in the range 4 to 6.

^4^ https://www.youtube.com/watch?v=lxRFeBr-KK4

## References

[ref1] Albarracín D. Mitchell A. L. (2004). The role of defensive confidence in preference for proattitudinal information: how believing that one is strong can sometimes be a defensive weakness. Pers. Soc. Psychol. Bull. 30, 1565–1584. doi: 10.1177/0146167204271180, PMID: 15536240 PMC4803283

[ref2] Andreu-Sánchez C. Martín-Pascual M. Á. Gruart A. Delgado-García J. M. (2021a). Viewers change eye-blink rate by predicting narrative content. Brain Sci. 11:422. doi: 10.3390/brainsci11040422, PMID: 33810422 PMC8065395

[ref3] Andreu-Sánchez C. Martín-Pascual M. A. Gruart A. Delgado-García J. M. (2021b). The effect of media professionalization on cognitive neurodynamics during audiovisual cuts. Front. Syst. Neurosci. 15:598383. doi: 10.3389/fnsys.2021.598383, PMID: 33584210 PMC7876408

[ref4] Brannon L. A. Tagler M. J. Eagly A. H. (2007). The moderating role of attitude strength in selective exposure to information. J. Exp. Soc. Psychol. 43, 611–617. doi: 10.1016/j.jesp.2006.05.001, PMID: 30198374

[ref5] Burnham K. P. Anderson D. R. (2004). Multimodel Inference. Sociol. Methods Res. 33, 261–304. doi: 10.1177/0049124104268644

[ref6] Cacioppo J. T. Petty R. E. (1982). The need for cognition. J. Pers. Soc. Psychol. 42, 116–131. doi: 10.1037/0022-3514.42.1.116

[ref7] Cacioppo J. T. Petty R. E. Feinstein J. A. Jarvis W. B. G. (1996). Dispositional differences in cognitive motivation: the life and times of people varying in need for cognition. Psychol. Bull. 119, 197–253. doi: 10.1037/0033-2909.119.2.197

[ref8] Cacioppo J. T. Petty R. E. Kao C. F. Rodriguez R. (1986). Central and peripheral routes to persuasion: an individual difference perspective. J. Pers. Soc. Psychol. 51, 1032–1043. doi: 10.1037/0022-3514.51.5.1032

[ref9] Cameron K. A. Jacks J. Z. O’Brien M. E. (2002). An experimental examination of strategies for resisting persuasion. Curr. Res. Soc. Psychol. 7, 205–224.

[ref10] Crano W. D. Crislin R. (2006). Attitudes and persuasion. Annu. Rev. Psychol. 57, 345–374. doi: 10.1146/annurev.psych.57.102904.190034, PMID: 16318599

[ref11] DeCarlo L. T. (1998). Signal detection theory and generalized linear models. Psychol. Methods 3, 186–205. doi: 10.1037/1082-989X.3.2.186

[ref12] Dodd M. D. Balzer A. Jacobs C. M. Gruszczynski M. W. Smith K. B. Hibbing J. R. (2012). The political left rolls with the good and the political right confronts the bad: connecting physiology and cognition to preferences. Philosoph Transact R Soc B Biol Sci 367, 640–649. doi: 10.1098/rstb.2011.0268, PMID: 22271780 PMC3260844

[ref13] Dorr M. Martinetz T. Gegenfurtner K. R. Barth E. (2010). Variability of eye movements when viewing dynamic natural scenes. J. Vis. 10, 1–28. doi: 10.1167/10.10.28 20884493

[ref14] Eagly A. H. Chaiken S. (1995). “Attitude strength, attitude structure, and resistance to change” in Attitude strength: Antecedents and consequences. eds. Petty R. E. Krosnick J. A. (Mahwah, NJ: Lawrence Erlbaum Associates Inc), 413–432.

[ref15] Fletcher C. R. Chrysler S. T. (1990). Surface forms, textbases, and situation models: recognition memory for three types of textual information. Discourse Process. 13, 175–190. doi: 10.1080/01638539009544752

[ref16] Haddock G. Maio G. R. Arnold K. Huskinson T. (2008). Should persuasion be affective or cognitive? The moderating effects of need for affect and need for cognition. Pers. Soc. Psychol. Bull. 34, 769–778. doi: 10.1177/0146167208314871, PMID: 18344496

[ref17] Haugtvedt C. P. Petty R. E. (1992). Personality and persuasion: need for cognition moderates the persistence and resistance of attitude changes. J. Pers. Soc. Psychol. 63, 308–319. doi: 10.1037/0022-3514.63.2.308

[ref18] Hollingworth A. Henderson J. M. (2002). Accurate visual memory for previously attended objects in natural scenes. J. Exp. Psychol. Hum. Percept. Perform. 28:113. doi: 10.1037/0096-1523.28.1.113

[ref19] Hutson J. P. Smith T. J. Magliano J. P. Loschky L. C. (2017). What is the role of the film viewer? The effects of narrative comprehension and viewing task on gaze control in film. Cognit Res Principles Implic 2:46. doi: 10.1186/s41235-017-0080-5, PMID: 29214207 PMC5698392

[ref20] Jacks J. Z. Cameron K. A. (2003). Strategies for resisting persuasion. Basic Appl. Soc. Psychol. 25, 145–161. doi: 10.1207/S15324834BASP2502_5

[ref21] Jacks J. Z. Devine P. G. (2000). Attitude importance, forewarning of message content, and resistance to persuasion. Basic Appl. Soc. Psychol. 22, 19–29. doi: 10.1207/S15324834BASP2201_3

[ref22] Knobloch-Westerwick S. Mothes C. Polavin N. (2020). Confirmation bias, ingroup bias, and negativity bias in selective exposure to political information. Commun. Res. 47, 104–124. doi: 10.1177/0093650217719596

[ref23] Krosnick J. A. Boninger D. S. Chuang Y. C. Berent M. K. Carnot C. G. (1993). Attitude strength: one construct or many related constructs? J. Pers. Soc. Psychol. 65, 1132–1151. doi: 10.1037/0022-3514.65.6.1132, PMID: 37515006

[ref24] Krosnick J. A. Petty R. E. (1995). “Attitude strength: an overview” in Attitude strength: Antecedents and consequences. eds. Petty R. E. Krosnick J. A. (Mahwah, NJ: Lawrence Erlbaum Associates Inc), 1–24.

[ref25] Langley M. M. Cleary A. M. Kostic B. N. Woods J. A. (2008). Picture recognition without picture identification: a method for assessing the role of perceptual information in familiarity-based picture recognition. Acta Psychol. (Amst) 127, 103–113. doi: 10.1016/j.actpsy.2007.03.001, PMID: 17434434

[ref26] Loftus G. R. (1972). Eye fixations and recognition memory for pictures. Cogn. Psychol. 3, 525–551. doi: 10.1016/0010-0285(72)90021-7, PMID: 37455314

[ref27] Loschky L. C. Larson A. M. Magliano J. P. Smith T. J. (2015). What would jaws do? The tyranny of film and the relationship between gaze and higher-level narrative film comprehension. PLoS One 10, 1–23. doi: 10.1371/journal.pone.0142474 PMC4659561 26606606

[ref28] Maki A. Raimi K. T. (2017). Environmental peer persuasion: how moral exporting and belief superiority relate to efforts to influence others. J. Environ. Psychol. 49, 18–29. doi: 10.1016/j.jenvp.2016.11.005

[ref29] Mandler G. (2008). Familiarity breeds attempts: a critical review of dual-process theories of recognition. Perspect. Psychol. Sci. 3, 390–399. doi: 10.1111/j.1745-6924.2008.00087.x, PMID: 26158957

[ref30] Nakano T. Yamamoto Y. Kitajo K. Takahashi T. Kitazawa S. (2009). Synchronization of spontaneous eyeblinks while viewing video stories. Proc. Biol. Sci. R. Soc. 276, 3635–3644. doi: 10.1098/rspb.2009.0828 PMC2817301 19640888

[ref31] O’Dea C. J. Castro Bueno A. M. Saucier D. A. (2018). Social vigilantism and the extremity, superiority, and defense of attitudes toward climate change. Personal. Individ. Differ. 130, 83–91. doi: 10.1016/j.paid.2018.03.040

[ref32] Peltier J. W. Schibrowsky J. A. (1994). Need for cognition, advertisement viewing time and memory for advertising stimuli. Adv. Consum. Res. 21, 244–250.

[ref33] Pertzov Y. Avidan G. Zohary E. (2009). Accumulation of visual information across multiple fixations. J. Vis. 9, 2.1–2.12. doi: 10.1167/9.10.2, PMID: 19810783

[ref34] Petty R. E. Wegener D. T. (1998). “Attitude change: multiple roles for persuasion variables” in The handbook of social psychology. eds. Gilbert D. Fiske S. Lindzey G.. 4th ed (New York: McGraw-Hill)

[ref35] Pomerantz E. M. Chaiken S. Tordesillas R. S. (1995). Attitude strength and resistance processes. J. Pers. Soc. Psychol. 69, 408–419. doi: 10.1037/0022-3514.69.3.408, PMID: 7562388

[ref36] Raimi K. T. Jongman-Sereno K. P. (2020). General belief superiority (GBS): personality, motivation, and interpersonal relations. Self Identity 19, 546–571. doi: 10.1080/15298868.2019.1640785

[ref37] Raimi K. T. Leary M. R. (2014). Belief superiority in the environmental domain: attitude extremity and reactions to fracking. J. Environ. Psychol. 40, 76–85. doi: 10.1016/j.jenvp.2014.05.005

[ref38] Ryu S. Vargas P. (2021). Product visuals and consumers’ selective exposure: the role of thought generation and cognitive motivation. J. Mark. Commun. 27, 780–798. doi: 10.1080/13527266.2021.1923556

[ref39] Saucier D. A. Webster R. J. (2010). Social vigilantism: measuring individual differences in belief superiority and resistance to persuasion. Pers. Soc. Psychol. Bull. 36, 19–32. doi: 10.1177/0146167209346170, PMID: 19776422

[ref40] Saucier D. A. Webster R. J. Hoffman B. H. Strain M. L. (2014). Social vigilantism and reported use of strategies to resist persuasion. Personal. Individ. Differ. 70, 120–125. doi: 10.1016/j.paid.2014.06.031

[ref41] Saucier D. A. Webster R. J. O’Dea C. J. Miller S. S. (2017). “The role of individual differences in inciting anger and social action” in Understanding angry groups: Multi-disciplinary perspectives on their motivations and effects on society. eds. Cloninger S. C. Leibo S. A. (Santa Barbara, CA: Praeger), 3–27.

[ref42] Schmalhofer F. Glavanov D. (1986). Three components of understanding of a programmer's manual: verbatim, propositional, and situational representations. J Memory Lang 25, 279–294. doi: 10.1016/0749-596X(86)90002-1

[ref43] Skitka L. J. Bauman C. W. Sargis E. G. (2005). Moral conviction: another contributor to attitude strength or something more? J. Pers. Soc. Psychol. 88, 895–917. doi: 10.1037/0022-3514.88.6.895, PMID: 15982112

[ref44] Smith T. J. Mital P. K. (2013). Attentional synchrony and the influence of viewing task on gaze behavior in static and dynamic scenes. J. Vis. 13:16. doi: 10.1167/13.8.16, PMID: 23863509

[ref45] Tatler B. W. Gilchrist I. D. Land M. F. (2005). Visual memory for objects in natural scenes: from fixations to object files. Q. J. Exp. Psychol. 58, 931–960. doi: 10.1080/02724980443000430, PMID: 16194942

[ref46] Tsfati Y. Cappella J. N. (2005). Why do people watch news they do not trust? The need for cognition as a moderator in the association between news media skepticism and exposure. Media Psychol. 7, 251–271. doi: 10.1207/S1532785XMEP0703_2, PMID: 32646827

[ref47] Van Dijk T. A. Kintsch W. (1983). Strategies of discourse comprehension. New York: Academic Press.

[ref48] Visser P. S. Bizer G. Y. Krosnick J. A. (2006). Exploring the latent structure of strength-related attitude attributes. Adv. Exp. Soc. Psychol. 38, 1–67. doi: 10.1016/S0065-2601(06)38001-X

[ref49] Visser P. S. Krosnick J. A. Simmons J. P. (2003). Distinguishing the cognitive and behavioral consequences of attitude importance and certainty: a new approach to testing the common-factor hypothesis. J. Exp. Soc. Psychol. 39, 118–141. doi: 10.1016/S0022-1031(02)00522-X

[ref50] Westerwick A. Johnson B. K. Knobloch-Westerwick S. (2017). Confirmation biases in selective exposure to political online information: source bias vs. content bias. Commun. Monogr. 84, 343–364. doi: 10.1080/03637751.2016.1272761

[ref51] Wright D. B. Horry R. Skagerberg E. M. (2009). Functions for traditional and multilevel approaches to signal detection theory. Behav. Res. Methods 41, 257–267. doi: 10.3758/BRM.41.2.257, PMID: 19363166

[ref52] Zelinsky G. J. Loschky L. C. (2005). Eye movements serialize memory for objects in scenes. Percept. Psychophys. 67, 676–690. doi: 10.3758/BF03193524, PMID: 16134461

[ref53] Zuwerink J. R. Devine P. G. (1996). Attitude importance and resistance to persuasion: It’s not just the thought that counts. J. Pers. Soc. Psychol. 70, 931–944. doi: 10.1037/0022-3514.70.5.931

