# Supplementary material for: The role of individual differences in resistance to persuasion on memory for political advertisements
Source: Front Psychol. 2023 Aug 9;14:1196209. doi: 10.3389/fpsyg.2023.1196209 (PMC10445487; doi:10.3389/fpsyg.2023.1196209)
Supplement: Supplementary file 1 [file Data_Sheet_1.docx]

**Supplementary Materials: Pilot Studies**

We conducted two pilot studies: one pilot study to develop the arguments to be used in the videos, and a second pilot study to evaluate the qualities of our political advertisement videos.

**Pilot Study 1:** **Argument Development**

We carefully developed the arguments used in the abortion advertisements and the debate. First, to identify common arguments on each side of the abortion issue, we created a pool of arguments by collecting abortion debates and advertisements. We identified the most common arguments from this pool. Next, we identified parallel arguments on each side of the abortion debate. We then pilot tested these parallel arguments with participants on both the pro-life and pro-choice sides of the abortion issue, having participants rate the arguments for their strength, persuasiveness, valence, and clarity (on 1 to 9 scales). Participants were classified as pro-life or pro-choice based on their responses to items measuring abortion attitudes adapted from Bumpass (1997) (e.g., “It should be possible for a woman to obtain a legal abortion if she wants one for any reason”) and Cook, Jelen, and Wilcox (1993) (e.g., “Public hospitals (funded by taxpayers) should be banned from performing abortions”). These items were assessed on a scale from 1 (*Strongly Disagree*) to 9 (*Strongly Agree*). Appropriate items were reverse-scored, and all items were averaged, such that participants who scored in the lower third of the scale were classified as pro-choice, and participants who scored in the upper third of the scale were classified as pro-life. Of the 19 participants we classified as pro-choice, 5 were men, 13 were women, and 1 identified as non-binary. Of the 16 participants we classified as pro-life, 7 were men, and 9 were women. The average age across both groups was 21.8 (*SD* = .85).

We selected arguments for use in the next phase of our studies based on participants’ mean ratings of strength, persuasiveness, valence, and clarity being above the midpoint for individuals who agreed with the argument (i.e., pro-choice participants who viewed pro-choice arguments (*n* = 9) and pro-life participants who viewed pro-life arguments (*n* = 6)), and below the midpoint for individuals who disagreed with it (i.e., pro-choice participants who viewed pro-life arguments (*n*  = 10) and pro-life participants who viewed pro-choice arguments (*n* = 10), and this difference in means (between those who agreed with the argument and those who did not) had to be statistically significant. Additionally, so that participants in our future studies would understand the arguments regardless of their views, we selected arguments that *both* pro-choice and pro-life participants rated above the midpoint on clarity and that had *no* significant difference in mean ratings of clarity between pro-life and pro-choice participants.

Given these criteria, we did not retain completely matching paired arguments for both the pro-life and pro-choice. However, it was more important that the arguments fit the above criteria than that they made perfectly matching opposing pairs for every issue of the abortion debate. Yet, three of the final five pairs of arguments (Table S1; arguments 1-3) from each side did address the same sets of issues of the debate. The non-matched arguments were rated as clearly stated by both sides, and rated as positive, strong, and persuasive by participants favoring that side of the debate.

We also created composite variables of the argument ratings for all five pro-choice arguments in the first column of Table S1 (i.e., the average of all five arguments’ strength ratings, the average of all five arguments’ persuasiveness ratings, etc.). We did the same for the five pro-life arguments in the second column of Table S1. We then compared the differences in each composite rating between pro-choice and pro-life participants. For the composites of all five pro-choice arguments, consistent with our criteria, there were significant differences (*p*s ≤ .003) between pro-choice and pro-life participants’ ratings of strength, persuasion, agreement, and valence. Also consistent with our criteria, the average clarity ratings on all five pro-choice arguments were not significantly different between pro-choice and pro-life participants. This confirms that the five pro-choice arguments were good choices for use in our studies.

For the composites of all five pro-life arguments, consistent with our criteria, there were significant differences (*p*s ≤ .01) between pro-life and pro-choice participants’ ratings of strength, persuasion, agreement, and valence. *Inconsistent* with our criteria, the average clarity ratings on all five pro-life arguments *was* significantly different between pro-life and pro-choice participants (*p* = .008, *M*_pro-life_ = 6.83, *M*_pro-choice_ = 4.16). This difference is likely driven by the first and fifth arguments in the pro-life column (Table 1). We speculate this difference in clarity ratings may be a result of either 1) bias playing a bigger role for pro-choice than for pro-life participants; or 2) though the pro-life arguments were clearly worded, the first and fifth arguments seem a bit more complex, so raters may have felt wanted more explanation, which came through in their ratings. Given these possible explanations, we were confident that these five pro-life arguments were appropriate for use, given that they met all other criteria.

Table S1. *Arguments Selected in Pilot Study 1*

|  | **Pro-Choice** | **Pro-Life** |
| --- | --- | --- |
| 1 | The point at which life begins has not been established, and since a fetus cannot survive outside the womb prior to 24 weeks, it cannot be treated as a separate life in need of equal protection under the law. | Life begins at conception/fertilization, and as such a fetus should be treated as a separate life in need of equal protection under the law. * |
| 2 | Women’s right to choose is protected by the constitutional right to privacy. | The legal system may restrict individuals’ rights to protect the innocent. |
| 3 | Women of all ages choose to have abortions and most do so after careful consideration of their circumstances. | Women who choose to have an abortion are often young and most do not consider the repercussions. |
| 4 | Forcing a woman to carry a pregnancy to term against her will is unethical. | Even legal abortions are not safe, and may result in later health risks (e.g., ectopic pregnancy, miscarriage, breast cancer). |
| 5 | The rights of the fetus do not outweigh the rights of the woman to choose. | Social support services are available for children, making abortion unnecessary in light of mothers’ financial concerns. * |

*Note:* The two arguments with a * symbol in the pro-life column did not quite meet our criteria for clarity, because pro-choice participants rated them below the midpoint. However, because they did meet all other qualifications, and seemed clearly worded to us, they were retained.

**Video Development and Pilot Study 2: Political advertisements**

After identifying the arguments to be used in the study in Pilot Study 1, we created videos using these arguments were developed for use in our experiments.

**Videos.** The videos shown to our participants included a non-controversial advertisement (control video), Pro-life advertisement, Pro-choice advertisement, and a practice video to give participants an idea of what they would be doing in the study. All of these videos can be found at https://www.youtube.com/playlist?list=PL_cgKzrGl6Q1a2uTx8qwOXuJ8-Qbc05Cs. The abortion ad videos were developed specifically for this research, while the non-controversial and practice videos were found online. Although the videos were from different sources, the videos found online were chosen to match the format of the abortion advertisements created for the experiment.

**Abortion advertisements.** We created the Pro-life and Pro-choice abortion advertisements using matching formats. The advertisements used intertitles to present the arguments, and had imagery that by itself would be neutral, but when paired with the arguments would strengthen the arguments being presented. We wanted intertitles that were brief enough to be read quickly in a short video. Therefore, we revised the top four original arguments (see Table S1) to create briefer parallel arguments for each advertisement (see Table S2).

To create the advertisements, a collaborator and professional video editor created intertitles for the videos using the revised arguments. We created a single visual theme for both videos by focusing on the hands of an individual. In the Pro-Life video, the majority of hands were those of children doing things like playing with Play-Doh or holding fruit. In the Pro-Choice video, the hands were mostly adult hands doing things like searching on a computer or holding one’s face. Thus, the visual content in the ads was very similar, but not identical. We designed the Pro-life ad to show the positives of being a child, and the Pro-choice to show the difficulty of making the decision to have an abortion.

To inform the participants about the position the advertisement would take from the beginning, each of the ads started with an intertitle that stated the ad was paid for by either a Pro-life or Pro-choice group (e.g., “Kansans for Life”). The arguments were presented as intertitles throughout the rest of the videos. At the end of each video a final intertitle told viewers to either “Vote for Choice” (Pro-choice video) or to “Choose Life” (Pro-life video). Each video also included instrumental background music. The Pro-life video was 59 seconds long, and the Pro-choice video was 1 minute and 7 seconds. The Pro-choice video was longer, because the intertitle text was longer in the Pro-choice video, and to accommodate for this the intertitles were shown for slightly longer to ensure participants had time to read the full text.

Table S2. *Simplified Arguments for Abortion Advertisements*

|  | **Pro-Choice** | **Pro-Life** |
| --- | --- | --- |
| 1 | Women today have the right to accomplish anything | Innocent lives should be protected |
| 2 | Women of all ages choose to have an abortion | Abortion is irresponsible and unsafe |
| 3 | There are many reasons for choosing an abortion | Life begins at conception |
| 4 | The rights of the fetus should not outweigh a woman’s rights | Life should be given a chance |

Participants (*N* = 117 college students ages 17 to 24, *M* = 18.86, *SD* = 1.26, 88% White, 72% female) watched the six short video advertisements in a randomized order: a) a pro-choice video, b) a pro-life video, c) a drunk-driving video, d) a social media video, e) a walking for health video, and f) a disabilities video. Participants evaluated each video by indicating their levels of agreement with eight items (measured on 1 = *Disagree Very Strongly*, to 9 = *Agree Very Strongly*, rating scales): 1) *How strongly do you agree with the overall message of the video?*, 2) *How positive was the overall message of the video?*, 3) *How controversial was the overall message of the video?*, 4) *How persuasive was the overall message of the video?*, 5) *How strong was the overall message of the video?*, 6) *How clear was the overall message of the video?*, 7) *How good were the production values (i.e., the combined technical qualities) of the video?*, and 8) *How easy was it to follow the overall message of the video?* Prior to watching the videos, participants indicated their attitudes about abortion on a single item: *On the issue of abortion, I am:* with response options ranging from 1 = *Very Strongly Pro-Choice*, to 9 = *Very Strongly Pro-Life* (five distractor items were also included that measured participants’ general attitudes about the other issues presented in the videos that were unrelated to abortion, e.g., drunk driving, walking for health, etc.). To simplify our analyses, participants who scored a 1 to 3 on the abortion attitudes item were coded as Pro-Choice (*n* = 36), participants who scored a 4 to 6 were coded as Neutral/Ambivalent (*n* = 35), and participants who scored a 7 to 9 were coded as Pro-Life (*n* = 46).

Table 3 displays the results of separate 2 (video: pro-choice, pro-life) x 3 (abortion attitudes: pro-choice, neutral/ambivalent, pro-life) mixed factorial analysis of variance (ANOVA) tests of participants’ evaluations of the pro-choice and pro-life videos. Unsurprisingly, there were significant differences in participants’ evaluations of the pro-life and pro-choice videos that were largely accounted for by participants’ attitudes about abortion. For our measures of agreement and persuasiveness, pro-life participants gave the pro-life video higher ratings than the pro-choice video, and pro-choice participants rated the pro-choice video higher than the pro-life video. For three of the video evaluation measures (clarity of the message, easy to follow, and production quality) pro-choice participants rated the pro-life video higher than the pro-choice video, but while these differences were in the same direction as the ratings from the pro-life participants, the differences were significantly stronger for pro-life participants compared to the pro-choice participants. Participants who were relatively neutral or ambivalent on the issue of abortion only showed differences in their ratings of how easy it was to follow the overall message of the video, such that they rated the pro-life video higher than the pro-choice video, but no significant differences between the two videos were found on the other seven video evaluation measures*.* Furthermore, participants’ ratings of the controversial nature of the video did not differ between the two videos, nor did their ratings of the two videos interact with participants’ attitudes about abortion. In summary, the differences in participants’ evaluations of the pro-choice and pro-life videos were largely a function of participants’ position on the issue of abortion. Furthermore, for participants who were relatively more neutral or ambivalent on the issue of abortion, their ratings did not significantly differ between videos on most of the video evaluation measures. Considering these data, and accounting for participants’ differing attitudes about abortion, we decided the pro-choice and pro-life videos were similar enough (in terms of their quality, persuasiveness, and controversial nature) to use as manipulations in our experiments.

Table S3. *Pro-Choice and Pro-Life Video Ratings by Participants’ Attitudes about Abortion*

| Item | Attitude about Abortion | Pro-Choice Video *M* (*SD*) | Pro-Life Video *M* (*SD*) | Diff. | *SE* | *p* |
| --- | --- | --- | --- | --- | --- | --- |
| *How strongly do you agree with the overall message of the video?* | Pro-Choice | 7.08 (1.80) | 3.36 (1.93) | 3.72 | .46 | < .001 |
|  | Neutral/Ambivalent | 5.12 (1.75) | 5.47 (1.21) | -0.35 | .48 | .460 |
|  | Pro-Life | 2.50 (2.14) | 8.50 (0.84) | -6.00 | .41 | < .001 |
| Video *F*(1, 113) = 11.38, *p* = .001,  = .09  Attitude *F*(2, 113) = 1.00, *p* = .370,  = .02  Video X Attitude *F*(2, 113) = 126.79, *p* < .001,  = .69 | | | | | | |
| *How positive was the overall message of the video?* | Pro-Choice | 5.08 (1.80) | 5.44 (2.26) | -0.36 | .40 | .369 |
|  | Neutral/Ambivalent | 5.18 (1.82) | 5.65 (1.30) | -0.47 | .41 | .256 |
|  | Pro-Life | 3.65 (2.02) | 7.63 (1.34) | -3.98 | .35 | < .001 |
| Video *F*(1, 113) = 50.82, *p* < .001,  = .31  Attitude *F*(2, 113) = 0.85, *p* = .430,  = .02  Video X Attitude *F*(2, 113) = 30.58, *p* < .001,  = .35 | | | | | | |
| *How controversial was the overall message of the video?* | Pro-Choice | 7.53 (2.08) | 7.36 (2.18) | 0.17 | .33 | .616 |
|  | Neutral/Ambivalent | 6.71 (2.55) | 6.71 (2.14) | < .001 | .34 | 1.00 |
|  | Pro-Life | 7.40 (2.13) | 7.17 (2.28) | 0.63 | .29 | .034 |
| Video *F*(1, 113) = 2.03, *p* = .157,  = .02  Attitude *F*(2, 113) = 1.92, *p* = .151,  = .03  Video X Attitude *F*(2, 113) = 1.10, *p* = .336,  = .02 | | | | | | |
| *How persuasive was the overall message of the video?* | Pro-Choice | 5.53 (1.78) | 5.11 (1.98) | 0.42 | .40 | .298 |
|  | Neutral/Ambivalent | 5.26 (1.78) | 5.62 (1.28) | -0.35 | .41 | .391 |
|  | Pro-Life | 3.87 (2.07) | 6.85 (1.74) | -2.98 | .35 | < .001 |
| Video *F*(1, 113) = 18.84, *p* < .001,  = .14  Attitude *F*(2, 113) = 0.07, *p* = .929,  = .001  Video X Attitude *F*(2, 113) = 23.08, *p* < .001,  = .29 | | | | | | |
| *How strong was the overall message of the video?* | Pro-Choice | 6.64 (1.68) | 6.36 (2.03) | 0.28 | .38 | .467 |
|  | Neutral/Ambivalent | 5.97 (1.96) | 6.26 (1.50) | -0.29 | .39 | .461 |
|  | Pro-Life | 4.83 (2.17) | 7.22 (1.58) | -2.39 | .34 | < .001 |
| Video *F*(1, 114) = 18.84, *p* < .001,  = .14  Attitude *F*(2, 114) = 0.07, *p* = .929,  = .001  Video X Attitude *F*(2, 114) = 23.08, *p* < .001,  = .29 | | | | | | |
| *How clear was the overall message of the video?* | Pro-Choice | 7.00 (2.27) | 7.83 (1.72) | -0.83 | .41 | .042 |
|  | Neutral/Ambivalent | 6.91 (2.13) | 7.00 (1.80) | -0.09 | .41 | .835 |
|  | Pro-Life | 5.87 (2.43) | 7.65 (1.88) | -1.78 | .36 | < .001 |
| Video *F*(1, 114) = 15.82, *p* < .001,  = .12  Attitude *F*(2, 114) = 1.60, *p* = .207,  = .03  Video X Attitude *F*(2, 114) = 4.94, *p* =.009,  = .08 | | | | | | |
| *How good were the production values (i.e., the combined technical qualities) of the video?* | Pro-Choice | 5.44 (2.10) | 6.17 (2.06) | -0.72 | .30 | .017 |
|  | Neutral/Ambivalent | 5.51 (1.96) | 5.89 (1.86) | -0.37 | .30 | .221 |
|  | Pro-Life | 4.76 (2.22) | 6.76 (1.95) | -2.00 | .26 | < .001 |
| Video *F*(1, 114) = 38.45, *p* < .001,  = .25  Attitude *F*(2, 114) = 0.20, *p* = .971,  = .001  Video X Attitude *F*(2, 114) = 9.56, *p* < .001,  = .14 | | | | | | |
| *How easy was it to follow the overall message of the video?* | Pro-Choice | 6.81 (2.50) | 7.61 (1.99) | -0.81 | .36 | .026 |
|  | Neutral/Ambivalent | 6.51 (2.24) | 7.29 (1.78) | -0.77 | .36 | .035 |
|  | Pro-Life | 6.00 (2.41) | 7.52 (1.81) | -1.52 | .32 | < .001 |
| Video *F*(1, 114) = 26.86, *p* < .001,  = .19  Attitude *F*(2, 114) = 0.59, *p* = .554,  = .01  Video X Attitude *F*(2, 114) = 1.64, *p* = .198,  = .03 | | | | | | |

*Note*. Error degrees of freedom differ between tests due to participants not responding to some of the items.

References

Cook, E. A. , Jelen, T. G., Wilcox, C. (1993). Measuring public attitudes on abortion: Methodological and substantive considerations. *Family Planning Perspectives, 25*(3), 118-121. doi: 10.2307/2136159

Bumpass, L. L. (1997). The measurement of public opinion on abortion: The effects of survey design. *Family Planning Perspectives, 29*(4), 177-180. doi: 10.2307/2953382
